# Molecular characterization of antimicrobial resistant non-typhoidal *Salmonella* from poultry industries in Korea

**DOI:** 10.1186/s13620-017-0095-8

**Published:** 2017-06-08

**Authors:** Jin Eui Kim, Young ju Lee

**Affiliations:** Department of Public Health, College of Veterinary Medicine, Kyungpook National University, Daegu, 41566 Republic of Korea

**Keywords:** Non-typhoidal Salmonella, Molecular characterization, Resistance

## Abstract

**Background:**

Antimicrobial resistant *Salmonella* strains are a direct threat to human health when this resistance interferes with treatment and an indirect threat when resistance can be transferred to other human pathogens. The objective of the present study was to characterize antimicrobial resistant non-typhoidal *Salmonella* (NTS) isolates recovered from poultry industries, including a description of genetic diversity and virulence profiles.

**Results:**

In total of 93 *Salmonella* isolates shown antimicrobial resistance to one or more drugs, all isolates exhibited common resistance to streptomycin, nalidixic acid and cephalothin but no ciprofloxacin resistance. Among 26 virulence gene profiling, 12 virulence genes, *invA, orgA, prgH, sopB*, *tolC, sipB*, *gatC, msgA, pagC, spiA, sifA,* and *sitC* were found in all antimicrobial-resistant NTS isolates. In comparing the data from ERIC-PCR clusters, virulence profiles and resistance profiles, some *Salmonella* isolates grouped into the same cluster were found to exhibit similar virulence and resistance patterns.

**Conclusions:**

Virulence profiling combined with ERIC-PCR offered a rapid approach to characterize antimicrobial-resistant NTS.

## Background

Non-typhoidal *Salmonella* (NTS) are major zoonotic food-borne pathogens causing gastroenteritis worldwide. The global burden of NTS infection is estimated to be 93.8 million cases of gastroenteritis each year [30]. In Korea, total of 9,472 human cases of food and waterborne salmonellosis were detected between 1998 and 2007 [25], and NTS strains are one of the most common causes of food poisoning in humans [23]. In many countries including Korea, NTS infections are associated with the consumption of contaminated food products, especially poultry meats and eggs [22, 37].

*Salmonella* virulence genes are present on the bacterial chromosome, plasmids, and prophages, and *Salmonella* pathogenicity islands (SPIs) play important roles in adhesion, invasion, intracellular survival, systemic infection, fimbrial expression, antibiotic resistance, toxin production, and Mg^2+^ and iron uptake [8]. For example, genes such as *invA, orgA, prgH, sipB* and *spaN* in SPI-1 encode a type 3 secretion system 1 (T3SS-1) which allows *Salmonella* to invade phagocytic and non-phagocytic cells. Genes such as *spiA* in SPI-2 encode a type 3 secretion system 2 (T3SS-2), which allows the survival and replication of *Salmonella* in host cells [27]. Other chromosomal genes such as *lpfA* and *pefA* encode fimbriae-associated proteins that are important for adherence [11]. Moreover, plasmidal genes such as *spvB* contribute to colonization of deeper tissues, among other functions [2]. The virulence potential of *Salmonella* determines the differences in pathogenicity among *Salmonella* serotypes.

The increase in antibiotic-resistant food-borne pathogens is also a major public health problem. A high rate of antimicrobial-resistance in *Salmonella* strains has been reported in Korea [5, 24]. Antimicrobial resistance and virulence of *Salmonella* strains play an important role in systemic infections with these pathogens [14].

Pulsed-field gel electrophoresis (PFGE) is the gold standard subtyping method used to assess relatedness among *Salmonella* strains from different sources [28]; however it is time consuming and labor intensive [16]. DNA-based fingerprinting techniques such as enterobacterial repetitive intergenic consensus (ERIC), repetitive extragenic palindromic (REP), and BOX repeat-based (BOXAIR) PCR methods are relatively easy to perform, rapid, and sensitive in discriminating between closely related strains [12, 16]. In recent years, the ERIC-PCR fingerprinting method has been used to confirm epidemiological relationships between various isolates, and this method has shown high discriminatory power [1, 32]. The objective of the present study was to characterize antimicrobial-resistant NTS isolates recovered from poultry industries, including a description of genetic diversity and virulence profiles.

## Methods

### Salmonella isolates

Ninty-three *Salmonella* isolates showing antimicrobial resistance to one or more drugs were tested in this study. All isolates were recovered from chilled chicken carcasses (*n* = 25) and chillers (*n* = 23) in chicken slaughter houses; chilled carcasses (*n* = 15) and chillers (*n* = 8) in duck slaughter houses; egg belts (*n* = 2), feeders (*n* = 1), feces (*n* = 5), dust (*n* = 3) and egg shells (*n* = 8) from layer farms; and raw shell eggs (*n* = 2) and egg contents (*n* = 1) from retail markets between 2008 and 2014 (Table 1). *Salmonella* isolates were serotyped according to the Kauffmann-White scheme following slide agglutination testing with *Salmonella*-specific O and H antisera (Difco, Detroit, MI).

**Table 1:** Distribution of 93 antimicrobial resistant *Salmonella* isolates derived from poultry industries in KoreaThe 17 chicken slaughter houses, 9 duck slaughter houses, 8 commercial layer farms and 3 retail markets were designated C1 ~ C17, D1 ~ D9, L1 ~ L8 and R1 ~ R3, respectively

*Salmonella* serovars	No	Source	Place	Year	Resistance pattern (n)
*S*. Agona	1	Feces	L6	2014	AMP-TE (1)
	1	Egg belts	L2	2013	CF-NA (1)
*S*. Bareilly	1	Egg belts	L3	2013	CF-NA-S (1)
	1	Egg shell	L2	2013	CF-NA (1)
	5	Egg shell	L3	2013	S (5)
	1	Feeder	L2	2013	S (1)
	1	Feces	L2	2013	S (1)
	1	Feces	L3	2013	S (1)
*S*. Binza	1	Chilled duck carcasses	D1	2011	AM-CF-S (1)
*S*. Braenderup	1	Egg shell	R1	2013	AM (1)
	1	Egg shell	R2	2013	AM (1)
	1	Feces	L5	2013	AM (1)
	1	Egg shell	L4	2013	AM (1)
	1	Dust	L5	2013	AM (1)
*S*. Coquilhativille	1	Chilled chicken carcasses	C15	2011	C-CF-S (1)
*S*. Enteritidis	1	Chicken chillers	C2	2008	NA (1)
	1	Chicken chillers	C3	2008	NA-S-TE (1)
	2	Chicken chillers	C4	2008	NA (2)
	2	Chicken chillers	C5	2008	AM-NA-S (1), AM-C-NA-S-TE (1)
	1	Chilled chicken carcasses	C1	2008	NA (1)
	5	Chilled chicken carcasses	C4	2008	NA (5)
	5	Chilled chicken carcasses	C5	2008	AM-NA-S (3), AM-CF-NA-S (2)
	1	Chicken chillers	C6	2011	AM-C-CF-NA-S (1)
	1	Chicken chillers	C7	2011	CF-NA (1)
	1	Chicken chillers	C8	2011	CF-NA-S (1)
	2	Duck chillers	D1	2010	TE (1), CF-S-TE (1)
	1	Duck chillers	D2	2011	AM-C-CF-NA (1)
*S*. Give	1	Chilled chicken carcasses	C14	2011	AMP-TE (1)
*S*. Hadar	2	Duck chillers	D3	2008	AM-CF-S-TE (1), AM-CF-KAN-S-TE (1)
	1	Duck chillers	D9	2011	CF-TE (1)
	1	Chilled duck carcasses	D1	2011	CF-S-TE (1)
	1	Chilled duck carcasses	D3	2008	KAN-S (1)
	2	Chilled duck carcasses	D4	2008	S-TE (2)
*S*. Hogton	1	Chilled duck carcasses	D4	2008	KAN-S-TE (1)
*S*. Infantis	1	Chilled chicken carcasses	C8	2011	CF-NA-S (1)
	1	Egg shell	L1	2014	NA (1)
*S*. Kortrijk	1	Chicken chillers	C15	2011	C (1)
*S*. Livingstone	1	Dust	L7	2014	NA (1)
*S*. London	1	Chilled duck carcasses	D3	2008	AM-CF-S-TE (1)
*S*. Malmoe	3	Chicken chillers	C9	2011	S (2), AM-C-CAZ-CF-CTX-NA-TE (1)
*S*. Mbandaka	2	Chilled duck carcasses	D5	2010	CF-S (1), S (1)
	1	Feces	L3	2013	CF (1)
*S*. Montevideo	1	Chicken chillers	C1	2008	NA (1)
	1	Chicken chillers	C9	2008	NA (1)
	1	Chicken chillers	C11	2011	CF-NA (1)
	1	Chicken chillers	C12	2011	CF-NA (1)
	3	Chilled chicken carcasses	C9	2008	NA (3)
	1	Chilled chicken carcasses	C10	2011	NA (1)
*S*. Newbrunswick	1	Chilled duck carcasses	D1	2011	S-TE (1)
*S*. Newport	2	Chicken chillers	C10	2011	NA (2)
	2	Chilled chicken carcasses	C10	2011	AM-CF-TE (1), CF-NA (1)
*S*. Ohio	1	Chilled duck carcasses	D4	2008	S-TE (1)
*S*. Orion	1	Chilled duck carcasses	D8	2011	CF-S (1)
*S*. Senftenberg	1	Chicken chillers	C9	2008	NA (1)
	1	Chicken chillers	C17	2011	CF-NA-S (1)
	1	Chilled chicken carcasses	C9	2008	NA (1)
	1	Chilled chicken carcasses	C13	2011	NA (1)
	1	Chilled chicken carcasses	C16	2011	AM (1)
	1	Dust	L8	2014	GEN-NA-S (1)
*S*. Takoradi	2	Chicken chillers	C9	2011	AM-CF-SXT (1)
*S*. Thomson	1	Chilled chicken carcasses	C7	2011	CF (1)
*S*. Trachau	1	Egg contents	R3	2013	CF (1)
*S*. Typhimurium	1	Chilled chicken carcasses	C10	2011	CTX (1)
	1	Duck chillers	D1	2010	CF-TE (1)
	1	Duck chillers	D3	2008	S-TE (1)
	1	Chilled duck carcasses	D2	2011	CF (1)
	1	Chilled duck carcasses	D6	2011	S-TE (1)
*S*. Wippra	1	Chilled duck carcasses	D6	2011	CF-S-TE (1)

### Antimicrobial susceptibility tests

Antimicrobial susceptibility profiles of the isolates were determined by the disk diffusion method [6]. Twelve antimicrobial agents (Difco, United States) were tested at the following concentrations: gentamicin (GM, 10 μg), kanamycin (K, 30 μg), cephalothin (CF, 30 μg), cefotaxime (CTX, 30 μg), ceftazidime (CAZ, 30 μg), chloramphenicol (C, 30 μg), ciprofloxacin (CIP, 5 μg), tetracycline (TE, 30 μg), ampicillin (AM, 10 μg), streptomycin (S, 10 μg), trimethoprim/sulfamethoxazole (SXT, 1.25/23.75 μg) and nalidixic acid (NA, 30 μg). An isolate was considered as multidrug-resistant (MDR) when exhibiting resistance to antimicrobials of at least three different classes [29]. *Escherichia coli* strain ATCC 25922 was used as a reference strain.

### Analysis of virulence genes

The DNA for all analyses was extracted by the boiling method [7], and 5 μl of DNA template (approximate 60 ng) was used in each PCR reaction. Primers details are presented in Table 2. To establish the reproducibility of the experiments, PCR reactions were performed twice. The amplified PCR products were visualized by electrophoresis on 1.5% agarose gels stained with ethidium bromide (0.5 μg/ml).

**Table 2:** Primers used in PCR for detection of virulence genes in antimicrobial resistant non-typhoidal *Salmonella*

Primer	Broad function (gene function)	Sequence (5’ to 3’)	Referance
*invA*	Host recognition/invasion (type III secretion system appratus)	F-CTGGCGGTGGGTTTTGTTGTCTTCTCTATT	Skyberg et al., (2006) [34]
		R-AGTTTCTCCCCCTCTTCATGCGTTACCC	
*orgA*	Host recognition/invasion (type III secretion system appratus)	F-TTTTTGGCAATGCATCAGGGAACA	Skyberg et al., (2006) [34]
		R-GGCGAAAGCGGGGACGGTATT	
*prgH*	Host recognition/invasion (type III secretion system appratus)	F-GCCCGAGCAGCCTGAGAAGTTAGAAA	Skyberg et al., (2006) [34]
		R-TGAAATGAGCGCCCCTTGAGCCAGTC	
*sopB*	Host recognition/invasion (type III secretion system appratus)	F-CGGACCGGCCAGCAACAAAACAAGAAGAAG	Skyberg et al., (2006) [34]
		R-TAGTGATGCCCGTTATGCGTGAGTGTATT	
*tolC*	Host recognition/invasion (outer membrane channel protein)	F-TACCCAGGCGCAAAAAGAGGCTATC	Skyberg et al., (2006) [34]
		R-CCGCGTTATCCAGGTTGTTGC	
*sopE*	Host recognition/invasion (invasion-associated secreted protein)	F-CATAGCGCCTTTTCTTCAGG	Suez et al., (2013) [35]
		R-ATGCCTGCTGATGTTGATTG	
*sseI*	Host recognition/invasion (type III secretion system effector protein)	F-CGCCATCATCAGTAACCGCC	Suez et al., (2013) [35]
		R-CTGCTGACCACATCCTCCC	
*ssek3*	Host recognition/invasion (type III secretion system effector protein)	F-TATCAATCTCAAATCATGG	Suez et al., (2013) [35]
		R-CGCGTTTATATCATACGTTTGC	
*sspH1*	Host recognition/invasion (type III secretion system effector protein)	F-GGTCACAGGACACGTTCTACG	Suez et al., (2013) [35]
		R-GCGCTTCTTCGTAATTTTCC	
*cdtB*	Host recognition/invasion (toxin-like protein)	F-ACAACTGTCGCATCTCGCCCCGTCATT	Skyberg et al., (2006) [34]
		R-CAATTTGCGTGGGTTCTGTAGGTGCGAGT	
*hlyE*	Host recognition/invasion (hemolysis-inducing protein)	F-GCGTGATTGAAGGGAAATTG	Suez et al., (2013) [35]
		R-CGAAAAGCGTCTTCTTACCG	
*lpfC*	Host recognition/invasion (fimbrial protein)	F-GCCCCGCCTGAAGCCTGTGTTGC	Skyberg et al., (2006) [34]
		R-AGGTCGCCGCTGTTTGAGGTTGGATA	
*pefA*	Host recognition/invasion (fimbial protein)	F-TAAGCCACTGCGAAAGATGC	Suez et al., (2013) [35]
		R-GCGTGAACTCCAAAAACCCG	
*tcfA*	Host recognition/invasion (fimbrial protein)	F-TCGCTATGTTTGCATGTGGT	Suez et al., (2013) [35]
		R-TTCAGGAACAGCCTCGAAGT	
*span*	Entry into nonphagocytic cells (type III secretion system appratus)	F-AAAAGCCGTGGAATCCGTTAGTGAAGT	Skyberg et al., (2006) [34]
		R-CAGCGCTGGGGATTACCGTTTTG	
*sipB*	Entry into nonphagocytic cells (translocation machinery component)	F-GGACGCCGCCCGGGAAAAACTCTC	Skyberg et al., (2006) [34]
		R-ACACTCCCGTCGCCGCCTTCACAA	
*spiA*	Survival within macrophage (outer membrane secretory protein)	F-CCAGGGGTCGTTAGTGTATTGCGTGAGATG	Skyberg et al., (2006) [34]
		R-CGCGTAACAAAGAACCCGTAGTGATGGATT	
*msgA*	Survival within macrophage (macrophage survival protein)	F-GCCAGGCGCACGCGAAATCATCC	Skyberg et al., (2006) [34]
		R-GCGACCAGCCACATATCAGCCTCTTCAAAC	
*pagC*	Survival within macrophage (virulence membrane protein)	F-CGCCTTTTCCGTGGGGTATGC	Skyberg et al., (2006) [34]
		R-GAAGCCGTTTATTTTTGTAGAGGAGATGTT	
*sodC*	Survival within macrophage (periplasmic Cu/Zn superoxide dismutase)	F-ATGACACCACAGGCAAAACG	Suez et al., (2013) [35]
		R-AGATGAACGATGCCCTGTCC	
*gatC*	Growth within host (PTS galactitol transporter subunit IIC)	F-ATTGGTATCGGCTTCGTGGG	Suez et al., (2013) [35]
		R-ATCCCCAGCCAGTATGAACC	
*spvB*	Growth within host (ADP-ribosylating toxin)	F-CTATCAGCCCCGCACGGAGAGCAGTTTTTA	Skyberg et al., (2006) [34].
		R-GGAGGAGGCGGTGGCGGTGGCATCATA	
*sitC*	Iron acquisition (permease)	F-CAGTATATGCTCAACGCGATGTGGGTCTCC	Skyberg et al., (2006) [34]
		R-CGGGGCGAAAATAAAGGCTGTGATGAAC	
*iron*	Iron acquisition (sidrophore)	F-ACTGGCACGGCTCGCTGTCGCTCTAT	Skyberg et al., (2006) [34]
		R-CGCTTTACCGCCGTTCTGCCACTGC	
sifA	Filamentous structure formation (secreted effector protein)	F-TTTGCCGAACGCGCCCCCACACG	Skyberg et al., (2006) [34]
		R-GTTGCCTTTTCTTGCGCTTTCCACCCATCT	
STM 2759	Putative dipeptice/oligopetide/nikel ABC-type transport systems	F-ACCATTTTCACCTGGGCTCC	Suez et al., (2013) [35]
		R-CGTTCAGGTTTTGTCGCTGG	

### ERIC-PCR fingerprints analysis

Genotyping of isolates was performed by ERIC-PCR using a pair of primers (F: 5’-ATG TAA GCT CCT GGG GAT TCA C-3’; R: 5’-AAG TAA GTG ACT GGG GTG AGC G-3’) [36]. The PCR reaction was performed using a lyophilized PCR master mix according to the manufacturer’s instructions. (AccuPower PCR PreMix, Bioneer, Korea). A thermocycler (Bio-Rad, Singapore) was programmed as follows: initial denaturation at 95 °C for 7 min, followed by 30 cycles of denaturation at 90 °C for 30 s, annealing at 52 °C for 1 min and extension at 65 °C for 8 min, and a final extention step at 65 °C for 16 min [39]. A negative control consisting of the same reaction mixture without a DNA template was included in each reaction. ERIC-PCR reactions were repeated at least twice for each isolate to determine the reproducibility of banding patterns. Data were analyzed using the software package BioNumerics 7.5 (Applied Maths, Keistraat, Belgium). A similarity dendrogram was constructed by the UPGMA method with a 1% tolerance limit and 1% optimization, using the DICE similarity coefficient. Clusters were identified based on an 80% similarity cut-off [10]. The discrimination index for ERIC-PCR was calculated using Simpon’s diversity index [19].

## Results

**Fig. 1:**
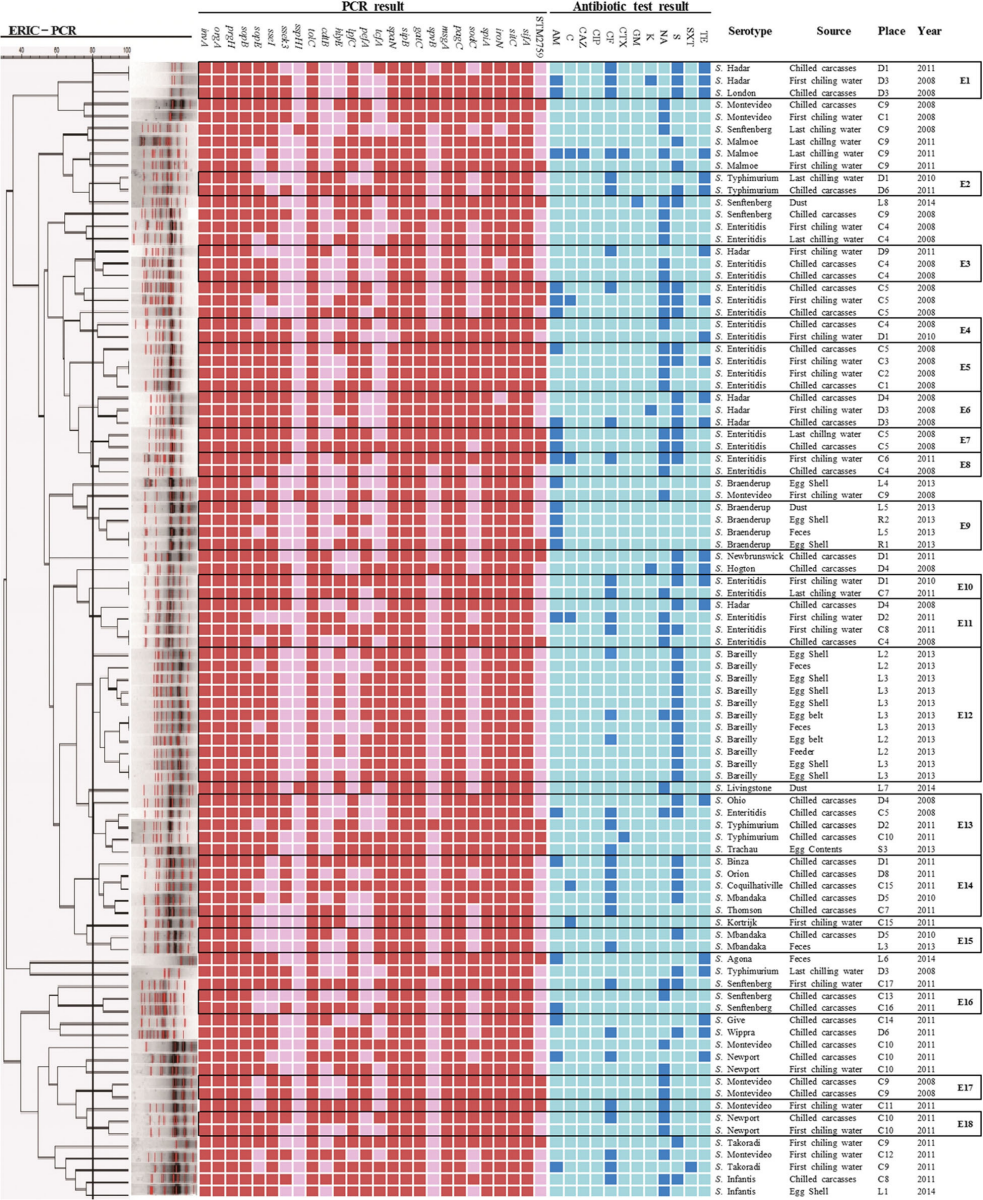
The ERIC-PCR analysis of the non-typhoidal Salmonella from Korean poultry industry is displayed using dendrograms generated by Bionumerics software. The *vertical line* shows the delineation level of 80%. The *red color* indicates the presence of the gene while the *pink color* indicates the absence of the gene. The *dark blue color* indicates resistance to the corresponding antibiotic while the *light blue color* indicates susceptibility. The 17 chicken slaughter houses, nine duck slaughter houses, eight commercial layer farms and three retail markets were designated C1 ~ C17, D1 ~ D9, L1 ~ L8, R1 ~ R3, respectively. AM ampicillin, C choloramphenicol, CAZ ceftazidime, CIP ciprofloxacin, CF cephalothin, CTX cefotaxime, GM gentamicin, K kanamycin, NA nalidixic acid, S streptomycin, STX trimethoprim/sulfamethoxazole, TE tetracycline

The results of ERIC-PCR, virulence gene profiling, and antimicrobial susceptibility test are summarized in Fig. 1. The 93 *Salmonella* isolates showed resistance to S (*n* = 46), NA (*n* = 42), CF (*n* = 34), AM (*n* = 24), TE (*n* = 22), C (*n* = 6), K (*n* = 3), CTX (*n* = 2), CAZ (*n* = 1), GM (*n* = 1), and SXT (*n* = 1). All isolates of *Salmonella* were susceptible to CIP. The 27 isolates (29%) showed multidrug resistance to more than three antibiotic classes.

From the virulence gene profiling, 12 virulence genes, *invA, orgA, prgH, sopB*, *tolC, sipB*, *gatC, msgA, pagC, spiA, sifA,* and *sitC* were found in all isolates and almost all of the isolates were positive for *spaN* (97%) and *iroN* (97%). However, the other 12 virulence genes, *sseI* (83%), *lpfC* (76%), *sopE* (70%), *hlyE* (60%), *pefA* (55%), *sodC* (48%), *tcfA* (48%), *ssek3* (43%), STM2759 (26%), *cdtB* (26%), *spvB* (20%) and *sspH1* (3%) were variably present in the isolates. Especially, *sspH1* gene was found in only 3 isolates, *S.* Montevideo, *S.* Livingstone *and S.* Senftenberg*.*

The ERIC-PCR analysis showed that 60 *Salmonella* isolates grouped into 18 clusters (E1 to E18) at 80% genetic similarity, whereas the remaining 33 isolates remained unclustered. The discrimination index of ERIC-PCR typing in this study was 0.974. Except for five clusters (E1, E3, E11, E13 and E14), isolates within each of the other 13 clusters belonged to the same serotype. Irrespective of their sources and places of isolation, all *S.* Bareilly isolates grouped into only one cluster (E12), whereas isolates of other serotypes could be found in different clusters, despite isolation from the same source and location. For example, 18 of 23 *S*. Enteritidis isolates grouped into eight clusters (E3, E4, E5, E7, E8, E10, E11 and E13) and the remaining five isolates of this serotype were not grouped. Two of 8 *S.* Montevideo isolates clustered into E17, and the remaining six isolates were not found in any cluster. Some of these *Salmonella* isolates representing the same serotype, sources, and location clustered separately.

## Discussion

Antimicrobial-resistant *Salmonella* strains are a direct threat to human health when this resistance interferes with treatment and an indirect threat when resistance can be transferred to other human pathogens [13]. In Korea, *S*. Enteritidis, *S*. Montevideo, *S*. Typhimurium is most common serotypes in poultry slaughter houses [4, 38], and the most frequently observed *Salmonella* serovars in the layer farms were *S*. Bareilly [20].

Cephems, quinolones and aminoglycosides have been widely used in the poultry industries in Korea [18, 24]. Therefore, *Salmonella* isolates tested in study showed consistent resistance to S, NA, and CF. Our finding showed that 29% among 93 antimicrobial resistance isolates were MDR. All MDR isolates recovered from chicken or duck slaughter houses. However, there was no MDR isolates recovered from layer farms or eggs of retail markets. Generally, *Salmonella* isolated from the broilers demonstrated greater MDR compared to those isolated from layer and eggs [21].

The ability of antimicrobial resistant NTS strains to cause invasive disease can be attributed to various virulence genes, and virulotyping rapidly allows the discrimination of isolates with diverse pathogenic potential [17]. In this study the genes-*invA, orgA, prgH, sopB*, *tolC, sipB*, *gatC, msgA, pagC, spiA, sifA,* and *sitC*, located in SPI-1, SPI-2, SPI-5, SPI-11, or others, were found in all antimicrobial-resistant NTS isolates. In addition, *spaN*, located in SPI-1, and *iroN*, located in an effector were highly conserved in the isolates. Similar findings were reported in a study of samples from both sick and healthy birds [34], clinical samples, and environmental samples from poultry houses [31], and free-living birds [26].

Recently some researchers have reported that typhoid-associated virulence genes (*cdt*B, *tcf*A, and *hly*E) in NTS serotypes of human and poultry origin are increasing [11, 26, 34]. NTS strains containing *cdtB*, *tcfA*, and *hlyE* genes were found in this study, and, to our knowledge, this is the first report to detect the presence of *cdtB* in the *S*. Hogton, *S*. Give, *S*. Newbrunswick, *S*. Thomson, *S*. Kortrijk, *S*. Coquilhativille, and *S*. Binza serotypes.

In this study, *lpfC*, one of three fimbrial genes, was more prevalent than the others, *pefA* and *tcfA*, which is consistent with previous studies [8, 15]. However, no *S*. Bareilly isolates tested in this study harbored the *lpfC* gene; whereas *tcfA* was presented in all *S*. Bareilly isolates, and *pefA* was highly conserved. Gong et al. [15] showed that the presence or absence of specific fimbrial genes in certain *Salmonella* serovars might have diagnostic value, as fimbrial genotypes can be used to determine certain *Salmonella* serotypes. Our results of fimbrial gene profiles are consistent with this report.

Molecular typing of *Salmonella* is vital to determining potential sources of infection and implementing effective epidemiological surveillance and control [9]. In this study, 60 out of 93 antimicrobial-resistant NTS isolates grouped into 18 ERIC-PCR clusters, and 33 isolates remained unclustered. This variability might be due to a difference in the sources of samples or in serotypes. In this study, the discrimination index of ERIC-PCR was 0.974. Based on a recommendation by Hunter et al. [19], a D value > 0.9 is desirable for good differentiation; our ability to discriminate between isolates was high. Based on these criteria, ERIC typing is useful for *Salmonella* typing, and our report showed that ERIC-PCR differentiated *Salmonella* strains indistinguishable to levels of heterogeneity of various serotypes.

The distribution of profiles among serotypes demonstrated that different serotypes showed similar fingerprinting patterns. These results are consistent with findings of Ranjbar et al. [33] who found that every Salmonella isolate had a unique fingerprinting but the serotypes were not grouped together in major branches.

In this study, the correlations among ERIC-PCR clusters, virulence profiles and resistance profiles were analyzed. We found that virulence genes and resistance profiles correlated with ERIC-PCR subtypes. Some isolates showed the same or similar virulotype or resistance pattern, irrespective of serotypes. The simultaneous presence of a resident virulence plasmid and resistance gene in the same bacterial cell has been reported in *Salmonella* [3]. Therefore, assessing the prevalence of virulence genes as well as the antibiotic resistance status in *Salmonella* serotypes would be useful to better understanding *Salmonella* pathogenicity.

## Conclusion

In conclusion, this study provides a molecular characterization of antimicrobial-resistant NTS from poultry industries in Korea. Virulence profiling combined with ERIC-PCR may offers a rapid approach to characterize antimicrobial-resistant NTS isolates. Therefore, determination of their definitive correlations will require future studies with isolates from various source of animal.

## Abbreviations

AM: Ampicillin
BOXAIR: BOX repeat-based
C: Chloramphenicol
CAZ: Ceftazidime
CF: Cephalothin
CIP: Ciprofloxacin
CLSI: Clinical and Laboratory Standards Institute
CTX: Cefotaxime
ERIC: Enterobacterial repetitive intergenic consensus
GM: Gentamicin
K: Kanamycin
MDR: Multidrug-resistant
NA: Nalidixic acid
NTS: Non-typhoidal *Salmonella*
PFGE: Pulsed-field gel electrophoresis
REP: Repetitive extragenic palindromic
S: Streptomycin
SPIs: *Salmonella* pathogenicity islands
SXT: Trimethoprim/sulfamethoxazole
T3SS-1: Type 3 secretion system 1
T3SS-2: Type 3 secretion system 2
TE: Tetracycline
